# Women’s Medical College of Baltimore

**Published:** 1893-04

**Authors:** Eugene F. Cordell

**Affiliations:** Baltimore


### Women's Medical College of Baltimore.

(From the Journal of the American Medical Association.)

In commencing the instruction in Latin of the students of the Woman's Medical College of this city, I gave the following reasons why as medical students they should acquire a knowledge of that language. I am glad to say that all the students of the college except three or four, and they having previously studied it elsewhere, have joined the class.

1. Because though called a "dead" language, and although not now spoken by any nation, it is not really dead, but flourishes with a perennial and ever increasing vigor.

2. Because of its wide and far-reaching influence on the structure and development of the languages of all civilized nations, especially those of Southern Europe.

3. Because the resources of our language do not suffice for the constantly needed new supply of words, for which we are compelled to resort to the "classics."

4. Because we cannot dispense, if we would, with the Latin that has been incorporated into our own language.

5. Because science and medicine are full of Latin terms and others are being constantly added to our scientific vocabulary.

6. Because of the aid it affords to the study of science and medicine and to the knowledge of the source and meaning of words.

7. Because even an elementary and imperfect knowledge of Latin will afford great assistance in your medical studies.

8. Because the study of Latin disciplines the mind, promotes habits of thought and attention, elevates the sentiments, imparts a scholarly tone, and furnishes one with noble examples, grand ideas and a magnificent literature.

9. Because no translation affords an adequate conception of the great classical authors, and besides if it did scarcely anyone would ever read it. Thus those who do not study the language itself are virtually cut off from all its benefits.

10. Because a classical and literary training is the most important preparation for the medical career.

11. Because a very respectable knowledge of Latin can be acquired at very little cost of time and study, perseverance being the chief element of success in its acquisition.

12. Because its requirement is in the line of elevation of the medical standard, and consequently of the standing of the medical profession.

13. Because this college in common with all respectable American medical colleges now requires it as a part of its preliminary requirements, in conformity with the regulations of the American Medical College Association.

14. Because, for the new students, the forms of its requirement are most liberal and accommodating, a year being allowed for preparation.

15. Because through the liberality of our Faculty the opportunity of acquirement is offered to all of you—whether first, second, third, or fourth year students, free of cost.

EUGENE F. CORDELL, M.D.

Baltimore, Oct. 18, 1892.

